# COVID-19 in patients with heart failure: the new and the old epidemic

**DOI:** 10.1136/postgradmedj-2020-138080

**Published:** 2020-07-30

**Authors:** Nicolò Sisti, Serafina Valente, Giulia Elena Mandoli, Ciro Santoro, Carlotta Sciaccaluga, Federico Franchi, Paolo Cameli, Sergio Mondillo, Matteo Cameli

**Affiliations:** Department of Medical Biotechnologies, Division of Cardiology, University of Siena, Siena, Italy; Department of Medical Biotechnologies, Division of Cardiology, University of Siena, Siena, Italy; Department of Medical Biotechnologies, Division of Cardiology, University of Siena, Siena, Italy; Department of Advanced Biomedical Sciences, Federico II University Hospital, Napoli, Italy; Department of Medical Biotechnologies, Division of Cardiology, University of Siena, Siena, Italy; Department of Medicine, Surgery and Neuroscience, Anesthesia and Intensive Care Unit, University of Siena, Siena, Italy; Department of Medicine, Surgery and Neurosciences, Respiratory Diseases and Lung Transplantation, University of Siena, Siena, Italy; Department of Medical Biotechnologies, Division of Cardiology, University of Siena, Siena, Italy; Department of Medical Biotechnologies, Division of Cardiology, University of Siena, Siena, Italy

**Keywords:** CARDIOLOGY, Heart failure

## Abstract

Severe acute respiratory syndrome coronavirus-2 (SARS-CoV-2) has spread in nearly 200 countries in less than 4 months since its first identification; accordingly, the coronavirus disease 2019 (COVID 2019) has affirmed itself as a clinical challenge. The prevalence of pre-existing cardiovascular diseases in patients with COVID19 is high and this dreadful combination dictates poor prognosis along with the higher risk of intensive care mortality. In the setting of chronic heart failure, SARS-CoV-2 can be responsible for myocardial injury and acute decompensation through various mechanisms. Given the clinical and epidemiological complexity of COVID-19, patiens with heart failure may require particular care since the viral infection has been identified, considering an adequate re-evaluation of medical therapy and a careful monitoring during ventilation.

## BACKGROUND

Since the beginning of December 2019, when first cases of pneumonia sustained by severe acute respiratory syndrome coronavirus 2 (SARS-CoV-2) were detected, the infection has reached the levels of a pandemic within 4 months, attesting 465.915 confirmed cases in the world and 21.031 deaths in 199 countries (WHO, 27 March 2020). The clinical features related to this ‘new’ coronavirus are similar to the ones caused by other coronaviruses known for past epidemics, such as SARS-CoV and Middle East respiratory syndrome (MERS)-CoV, although epidemiological data point at a lower mortality. The clinical characteristics of coronavirus disease 2019 (COVID-19) have rapidly increased interest and concerns. As described by the first analyses on the Chinese population, a severe clinical evolution of pneumonia, with a sustained risk of intensive care admission, intubation and death represents a recurrent in patients of advanced age or affected by chronic diseases.^1^ Chronic heart failure (CHF) involves more than 10% of population over 70 years in the developed countries, making it a known epidemic that is inevitably shattering with the one currently caused by SARS-CoV-2.^2^ The purpose of this review is to describe the actual evidences on COVID-19 in the population affected by CHF. Epidemiological characteristics, prognostic aspects and therapeutic implications are discussed according to the limited existing evidence from the first case reports in COVID-19 populations.

## HEART FAILURE IN THE REPORTED POPULATIONS

Shi *et al* reported that 4.1% (seventeen patients) of the COVID-19 population included presented CHF as pre-existing condition.^3^ In a different Chinese cohort, the occurrence of heart failure (HF) affected the 23% of the patients with COVID-19, but no information was provided about the pre-existing condition, chamber involved or type of dysfunction.^4^ To obtain deeper insight about the connection between CHF and COVID-19, the prevalence of two of the main determinants of HF, such as arterial hypertension (AH) and coronary artery disease (CAD), need to be explored in the infected population. In the study by Guan *et al*, among 1099 patients, nearly 18% had one or both conditions,^1^ while this percentage rises to 30–38% in the reports by Huang *et al* and Zhou *et al*, even though smaller cohorts were included in these studies.^4 5^ In an additional metanalysis based on eight studies with a total of 46 248 patients,^6^ the combined prevalence of AH and CAD was about 20% (17±7%, 95% CI 14% to 22% and 5±4%, 95% CI 4% to 7% for AH and CAD, respectively). The main registries focused on the Chinese population with CHF indicate a prevalence of AH ranging from 50% to 70% and of CAD from 50% to 60%,^7^ allowing us to have an estimation of the burden of the disease. If diabetes is also included, such results could be even underestimated. All the featured studies agree that chronic heart diseases, including CHF, are among the variables that might promote a severe phenotype of COVID-19 characterised by worse prognosis and increased mortality.

## INFECTION BY SARS-CoV-2 AND THE FAILING HEART

HF is a recognised vulnerability during respiratory viral infections, due to physiopathological mechanisms which are also involved in SARS-CoV-2 infection. Such mechanisms predispose to a decompensation of HF and to increasing arrhythmic and ischaemic risk.^8^ The inflammatory status and the production of cytokines secondary to the infection increase blood viscosity and coagulability, cause endothelial dysfunction, and promote electrolyte and haemodynamic imbalance.^9^ The advanced phases of the infection seem to be characterised by a cytokine storm whose profile seems to be similar to the one encountered in haemophagocytic lymphohistiocytosis secondary to viral infections, with a typical increase of interleukin (IL) 3, IL-6, IL-7, granulocyte-colony stimulating factor, interferon-γ inducible protein 10, monocyte chemoattractant protein 1, macrophage inflammatory protein 1-α, and tumour necrosis factor-α (TNF-α) and ferritin.^10^ As demonstrated for other viruses, this feature of SARS-CoV-2 could predispose to stress cardiomyopathy and cytokine-related myocardial dysfunction with consequent acute decompensation of CHF deteriorating subclinical pre-existing damage in well-compensated patients.^8^ Together with the inflammatory status, the respiratory failure caused by the viral infection can further aggravate the imbalance between the scarce supply of oxygen and the higher energy demand of the myocardium eliciting myocardial dysfunction.^11^ An additional aspect to be considered consisted in the myocardial damage induced by pulmonary hypertension, particularly involving the right chambers. Moreover, the use of elevated positive end-expiratory pressure during mechanical ventilation induces an increase in right ventricular afterload and wall stress, leading to a higher risk of further reducing cardiac output in the presence of a failing heart.^12^ Consistently with previous SARS- and MERS-epidemics, in patients with COVID-19, hypotension, tachycardia, bradycardia, cardiomegaly and arrhythmia are recurrent conditions recognized as predisposing to acute HF.^13^ Patients requiring intensive care show higher values of blood pressure than patients requiring standard care (145 mmHg vs 122 mmHg; p<0.001)^5^: on the other hand, an hypertensive profile seems to be reassuring regarding a minor risk of requiring inotropic support and developing cardiogenic shock.^14 15^ During the past coronavirus epidemics, some authors underlined a subclinical diastolic dysfunction of the left ventricle that seemed to be reversible on clinical recovery, while systolic dysfunction was associated with a higher need for mechanical ventilation.^16^ Mehra *et al* suggest that, in patients with pre-existing subclinical heart damage, during the early phases, when pulmonary complication and haemodynamic instability prevails, COVID-19 is associated with diastolic dysfunction, while systolic dysfunction subsequently upraises as a consequence of cytokine effect.^8^

## MARKERS OF MYOCARDIAL INJURY IN COVID-19

The myocardial injury, as expressed by the reported rise in troponin (Tn) during COVID-19,^3^ can be elicited by several mechanisms, besides the ones previously described (eg, systemic inflammation and hypoxia), that particularly concern patients with pre-existing cardiovascular disease. Among the most discussed features of SARS-CoV-2, its functional receptor, the aminopeptidase ACE2 (human ACE2), plays a central role, being overly expressed in patients with cardiovascular disease.^17^ Indeed, the viral infection of the cells, through the binding of ACE2, may set off direct myocardial damage, as it was demonstrated during the SARS outbreak.^18^ ACE2 expression in the heart is an essential regulator of function, and ACE2 knockout models are inclined to develop severe left ventricular dysfunction. SARS-CoV infection appears to downregulate ACE2, being a trigger to myocardial dysfunction.^19^ Even if a precise mechanism of cardiac injury is not identified, a direct role of the virus is suggested by autopsies in patients with myocarditis, which found viral genome by PCR in 35% of cases together with hypertrophy and low levels of ACE2 expression.^18^ Moreover, downregulation of ACE2 in cardiac muscle enhances TNF-α production and transforming growth factor-β signalling increasing local inflammatory response and fibrosis, respectively.^20 21^ The anti-inflammatory and antioxidant effects of ACE2 seem to oppose the deleterious effect of angiotensin II, especially when the renin–angiotensin–aldosterone system (RAAS) is upregulated, as it happens in AH, atherosclerosis and HF.^22^ As a consequence, alteration of ACE2 signalling pathways, determined by SARS-CoV-2 binding,^23^ may contribute to the poorer outcome of patients suffering from cardiovascular disease associated with the higher risk of developing severe conditions.^24 25^ In patients affected by both systolic and diastolic CHF, as well as acute decompensation, the increase in high-sensitivity Tn (hs-Tn) has a confirmed negative prognostic value.^26–^ ^28^ As confirmed in the report by Zhou *et al*,^4^ nearly 50% of ‘non-survivor’ patients present levels of hs-Tn >28 ng/mL with a maximal increase after 16 days from the onset of symptoms. Data regarding CHF emerged in a study on 416 patients with COVID-19 from the Renmin Hospital of Wuhan^3^ and divided at admission according to the presence of myocardial injury, indicated by hs-Tn blood levels above 99th percentile. In patients with myocardial injury, higher prevalence of CHF (15% vs 1.5%, p<0.01) and higher levels of N-terminal-pro-B-type natriuretic peptide(NT-proBNP) (median 1689 (698–3327) pg/mL vs 139 (51–335) pg/mL, p<0.001) were reported compared with those without myocardial injury. The levels of NT-proBNP >900 pg/mL on admission were associated with an increased, although not significant, mortality (HR 1.52; 95% CI 0.74 to 3.10; p=0.25). Myocardial injury showed to be a significant predictor of mortality in patients with COVID-19 (on admission HR 3.41 (95% CI 1.62 to 7.16) p<0.001) in a multivariate analysis after adjusting for different factors including NT-proBNP and cardiovascular disease such as CHF. To date, data are uncertain regarding long-term effects of myocardial injury during COVID-19. Most patients maintain a preserved ejection fraction which could suggest a positive prognosis, but details regarding the burden of fibrosis or indirect marker such as delayed enhancement are still lacking.^29^

## INSTRUMENTAL EXAMINATION: PNEUMONIA OR CONGESTION?

Among the first challenges following the spread of SARS-CoV-2 in patients with CHF, there was the need to distinguish the viral lung damage from acute pulmonary oedema through instrumental examination, enabling better prognostic stratification and therapeutic framing. Since chest X-ray is affected by low sensitivity in this context, integrated evaluation of both CT findings and lung ultrasound appear to be crucial. Zhu *et al* ^30^ highlighted that both clinical conditions can present ground glass area and thickened interlobular septa. In case of pulmonary oedema, these alterations are less numerous and more represented near the hila and dorsally, and are frequently associated with pleural effusion, cardiomegaly, pulmonary vein enlargement and rapid resolution after diuretic therapy. The lung ultrasound in patients with cardiogenic pulmonary oedema frequently shows a well-defined B-line pattern with a ‘white lung’ evolution in severe forms. The typical lung ultrasound findings in COVID-19 highlight in early phases irregular pleural lines, B-lines with an irregular distribution associated with limited areas of ‘white lung’. Such a pattern is inclined to spread consensually with the involvement of lung parenchyma, and it is associated with subpleural consolidation with or without air bronchogram. Some authors describe a C pattern in more advanced stages, when B-lines disappear anteriorly (because of ventilation support), with lateral and posterior subpleural consolidation.^31 32^

## THERAPEUTIC IMPLICATIONS

**Figure 1:**
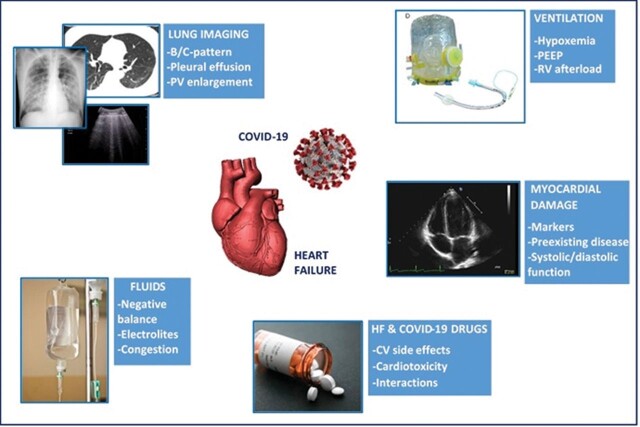
Findings suggestive of decompensation and variables to be considered in the management of patients with COVID-19 having a history of heart failure. CV, cardiovascular; COVID-19, coronavirus disease 2019; PEEP, pulmonary end-expiratory pressure; PV, pulmonary vein (Images by Reaper DZ on Pixabay and CDC on Unsplash).

The mechanism of action of ACE inhibitors (ACEIs) and angiotensin receptor blockers (ARBs) has been immediately correlated with SARS-CoV-2, given that the ACE2 enzyme is the viral functional receptor. Conflicting evidence exists about the ability of these drugs to increase the enzyme expression^33^; nevertheless, since there is no clear relationship between SARS-CoV-2 infections and these therapies, international communities have from the beginning recommended against the interruption of such drugs.^34^ One of the first studies to analyse the impact on prognosis of ACEIs and ARBs in patients with COVID-19 was performed by Huang *et al* who compared 20 patients with hypertension under ACEIs/ARBs and 30 patients with hypertension using other drugs. No significant difference was found among the two groups in the in-hospital mortality (p=0.265), time from onset to discharge or to negative test (p=0.541 and p=0.146 respectively), worsened chest CT during hospitalisation (p=0.450). Although Tn-I and NT-proBNP levels were lower in the ACEIs/ARBs group (0.01±0.01 vs 0.1±0.22; p=0.03 for Tn-I and 43.39 vs 263.05; p=0.04 for NT-proBNP), patients with markers above the pathological threshold did not show any significant difference between the two groups and, additionally, such difference between the two groups was not significant in patients aged over 65 years and under 65 years.^35^ Results from 42 patients with hypertension treated at the Shenzhen Third People’s Hospital (17 under ACEIs/ARBs and 25 under other drugs) first confirmed the beneficial clinical effect of renin–angiotensin system inhibition in COVID-19. In detail, ACEIs/ARBs group presented a less severe disease and lower levels of IL-6, a higher absolute number of CD3+ and CD8+ T cells, and a lower peak viral load during hospitalisation.^36^ A great interest is gathered around the protective effect of ACE2 on lung damage in COVID-19. In fact, the virus seems to cause a downregulation of ACE2, which determines an increased activity of angiotensin II with higher vascular permeability.^37^ Losartan, in particular, has already showed a protective role in different cases of ‘lung injury’, and specific trials are ongoing to determine its beneficial use in COVID-19.^38^ However, an important aspect to consider is the effect of RAAS inhibitors on the bradykinin levels: if on one hand no reason exists to interrupt therapy in patients without lung involvement, it can be reasonable when significant pneumonia and acute respiratory distress syndrome occur.^39^ The use of diuretics in patients with COVID-19 must be carefully monitored together with the administration of fluids, to maintain a negative fluid balance and to reduce the risk of an overlap between infective lung damage and cardiogenic pulmonary oedema. As reported in patients with CHF during other respiratory virus epidemics^40 41^ and later confirmed in the first reports on COVID-19 cases, acute kidney injury complicates 3–50% of severe pneumonia with an onset in the first 15 days, resulting as an important predictor of mortality.^1 4^ In patients with HF, antiviral therapy can have a detrimental impact on myocardial function and the risk of cardiotoxicity must be carefully evaluated. Possible interactions may occur between drugs used in CHF and the ones currently used for the management of COVID-19, which may determine combined cardioactive effects. In detail, digoxin clearance can be reduced by hydroxychloroquine or ritonavir. Hydroxychloroquine itself may have an arrhythmogenic effect in long-term users and tocilizumab can lead to a hypertensive profile.^11^ Although supported by small studies, clinicians in many countries have begun using the combination of azithromycin and hydroxychloroquine in the treatment of COVID-19 with a consequent risk of increased QTc prolongation.^42 43^ Structural heart disease together with electrolyte disturbances and hepatic/renal failure are all triggers of drug-induced torsade de pointes, making a close monitoring for these patients mandatory. History of long QT syndrome, baseline QTc >500 ms or QTc increase >60 ms should suggest dose adjustment or drug discontinuation.^44 45^ Finally, patients with HF who were affected by CAD seem to be more predisposed to plaque rupture during systemic inflammation throughout viral infections, confirming the importance of continuing the anti-ischaemic and plaque stabilisation therapy in this particular setting^46^  figure 1. Table 1 enlists some of the evidences to consider in the management of patients with HF who were affected by COVID-19 according to previously cited articles.

**Table 1:** Summary of the main evidences on clinical variables in patients with HF who were affected by COVID-19

Variable	Evidence
Left diastolic dysfunction	Often subclinical and reversible in early phases^8 16^
Left systolic dysfunction	Typical of forms with important cytokines release^8^ Associated with a higher need of mechanical ventilation^16^
Right heart dysfunction	Associated with secondary pulmonary hypertension^11^ Can be triggered by high positive end-expiratory pressure^12^
Troponin	Abnormal levels associated with pre-existing chronic heart failure^3^ Levels >28 ng/mL associated with “nonsurvivor” phenotypes^4^
NT-proBNP	Levels >900 pg/mL associated with increased mortality^3^ Increased in presence of myocardial injury^3^
ACEIs/ARBs	Associated with lower levels of troponin and NT-proBNP^35^ No difference in clinical course from patients not treated^35^ Associated with less severe disease, lower cytokines release, lower viral load^36^

ACEIs/ARBs, ACE inhibitors/angiotensin receptor blockers; COVID-19, coronavirus disease 2019; NT-proBNP, N-terminal-pro-B-type natriuretic peptide.

## CONCLUSIONS

The prevalence of CHF in the population susceptible to COVID-19 is significant and so is the prevalence of predisposing conditions which put infected patients at risk of developing HF. For this reason, promoting a thorough knowledge of the clinical implications and the prognostic impact of COVID-19 in this vulnerable category is a priority. In particular, the management of these patients needs to be set on the early detection of peculiar clinical and instrumental patterns, through a comprehensive cardiologic monitoring that allows the clinician to anticipate complications and to target therapeutic changes.

**Main messages**

- COVID-19 represents a clinical challenge in patients with heart failure.
- Heart damage is an important prognostic predictor of poor outcome.
- Therapeutic management of patients with heart failure must be carefully re-evaluated accordingly to the clinical condition due to the infection.

**Current research questions**

1. Does SARS-CoV-2 exerts a direct or indirect action on myocardial tissue?
2. Are there better and worse choices for heart failure drugs among patients with COVID-19?
3. Are there long-term implications of SARS-CoV-2 infection for patients with heart failure?

**Key references**

- W.-J. Guan, Z.-Y. Ni, Y. Hu, W.-H. Liang, C.-Q. Ou, J.-X. He, L. Liu, H. Shan, C.-L. Lei, D.S.C. Hui, B. Du, L.-J. Li, G. Zeng, K.-Y. Yuen, R.-C. Chen, C.-L. Tang, T. Wang, P.-Y. Chen, J. Xiang, S.-Y. Li, J.-L. Wang, Z.-J. Liang, Y.-X. Peng, L. Wei, Y. Liu, Y.-H. Hu, P. Peng, J.-M. Wang, J.-Y. Liu, Z. Chen, G. Li, Z.-J. Zheng, S.-Q. Qiu, J. Luo, C.-J. Ye, S.-Y. Zhu, N.-S. Zhong, China Medical Treatment Expert Group for COVID-19, Clinical Characteristics of Coronavirus Disease 2019 in China, *N. Engl. J. Med*. (2020) 1–13. https://doi.10.1056/NEJMoa2002032.
- Y.Y. Zheng, Y.T. Ma, J.Y. Zhang, X. Xie, COVID-19 and the Cardiovascular System, *Nat. Rev. Cardiol*. (2020). https://doi.10.1038/s41569-020-0360-5.
- K.J. Clerkin, J.A. Fried, J. Raikhelkar, G. Sayer, J.M. Griffin, A. Masoumi, S.S. Jain, D. Burkhoff, D. Kumaraiah, L. Rabbani, A. Schwartz, N. Uriel, Coronavirus Disease 2019 (COVID-19) and Cardiovascular Disease, *Circulation* 2019 (2020). https://doi.10.1161/CIRCULATIONAHA.120.046941.
- Z. Xu, L. Shi, Y. Wang, J. Zhang, L. Huang, C. Zhang, S. Liu, P. Zhao, H. Liu, L. Zhu, Y. Tai, C. Bai, T. Gao, J. Song, P. Xia, J. Dong, J. Zhao, F.S. Wang, Pathological findings of COVID-19 associated with acute respiratory distress syndrome, *Lancet Respir. Med*. 2600 (2020) 19–21. https://doi.10.1016/S2213-2600(20)30076-X.
- S. Shi, M. Qin, B. Shen, Y. Cai, T. Liu, F. Yang, W. Gong, X. Liu, J. Liang, Q. Zhao, H. Huang, B. Yang, C. Huang, Association of Cardiac Injury with Mortality in Hospitalized Patients with COVID-19 in Wuhan, China, *JAMA Cardiol*. (2020) 1–8.

**Multiple-choice questions**

1. The advanced phases of the infection by SARS-CoV-2
  - seem to be characterised by a cytokine storm
  - is similar to haemophagocytic linfoistyocytosis
  - is characterised by the increase of interleukin-3
2. In patients with myocardial injury,
  - higher prevalence of CHF is reported
  - NT-proBNP is reduced
  - levels of NT-proBNP >200 pg/mL on admission were associated with a poor prognosis
3. dose adjustment or drug discontinuation should be performed for
  - baseline QTc >500 ms
  - QTc increase >60 ms
  - baseline QTc >300 ms
4. Chronic heart failure (CHF) involves
  - more than 10% of population aged over 30 years
  - more than 10% of population aged over 70 years
  - more than 50% of population aged over 40 years
5. In patients with pre-existing subclinical heart damage,
  - COVID-19 is associated with diastolic dysfunction
  - systolic dysfunction upraises as a consequence of cytokine effect
  - diastolic function is usually preserved

**Answers**

(A) True (B) True (C) True
(A) True (B) False (C) False
(A) True (B) True (C) False
(A) False (B) True (C) False
(A) True (B) True (C) False
